# Comparative efficacy and acceptability of non-pharmacological interventions in fibromyalgia: Protocol for a network meta-analysis

**DOI:** 10.1371/journal.pone.0274406

**Published:** 2022-10-03

**Authors:** Mateus B. Souza, Rodrigo O. Mascarenhas, Laisa B. Maia, Letícia S. Fonseca, Hytalo J. Silva, Rutger M. J. de Zoete, James H. McAuley, Nicholas Henschke, Vinicius C. Oliveira

**Affiliations:** 1 Postgraduate Program in Rehabilitation and Functional Performance, Universidade Federal dos Vales do Jequitinhonha e Mucuri (UFVJM), Diamantina, Brazil; 2 Department of Physiotherapy, Universidade Federal dos Vales do Jequitinhonha e Mucuri (UFVJM), Diamantina, Brazil; 3 Postgraduate Program in Health Sciences, Universidade Federal dos Vales do Jequitinhonha e Mucuri (UFVJM), Diamantina, Brazil; 4 School of Allied Health Science and Practice, The University of Adelaide, Adelaide, South Australia, Australia; 5 Neuroscience Research Australia, University of New South Wales, Sydney, New South Wales, Australia; 6 Institute for Musculoskeletal Health, School of Public Health, The University of Sydney, Sydney, Australia; Thamar University, Faculty of Dentistry, YEMEN

## Abstract

**Introduction:**

Although several non-pharmacological interventions have been tested in the management of Fibromyalgia (FM), there is little consensus regarding the best options for the treatment of this health condition. The purpose of this network meta-analysis (NMA) is to investigate the comparative efficacy and acceptability of non-pharmacological interventions for FM, in order to assist clinical decision making through a ranking of interventions in relation to the most important clinical outcomes in these patients.

**Methods and analysis:**

We will perform a systematic search to identify randomised controlled trials of non-pharmacological interventions endorsed in guidelines and systematic reviews. Information sources searched will include major bibliographic databases without language or date restrictions (MEDLINE, Cochrane Library, EMBASE, AMED, PsycINFO and PEDro). Our primary outcomes will be pain intensity, patient-reported quality of life (QoL), and acceptability of treatment will be our secondary outcome. Risk of bias of the included trials will be assessed using the Cochrane risk of bias tool (RoB2). For each pairwise comparison between the different interventions, we will present mean differences (MDs) for pain intensity and QoL outcomes and Relative Risks (RRs) for acceptability, both with respective 95% confidence intervals (CIs). Initially, standard pairwise meta-analyses will be performed using a DerSimonian-Laird random effects model for all comparisons with at least two trials and then we will perform a frequentist NMA using the methodology of multivariate meta-analysis assuming a common heterogeneity parameter, using the mvmeta command and network suite in STATA. In the NMA, two different types of control group, such as placebo/sham and no intervention/waiting list will be combined as one node called “Control”. The competing interventions will be ranked using the P-score, which is the frequentist analogue of surface under the cumulative ranking curve (SUCRA) for the outcomes of interest at immediate- (intervention duration of up to 2 weeks), short- (over 2 weeks up to 12 weeks) and long-terms (over 12 weeks). The confidence in the results from NMA will be assessed using the Confidence in Network Meta‐analysis (CINeMA) framework.

**Ethics and dissemination:**

This work synthesises evidence from previously published studies and does not require ethics review or approval. A manuscript describing the findings will be submitted for publication in a peer-reviewed scientific journal.

**Registration:**

OSF (DOI: 10.17605/OSF.IO/7MS25) and registered in the PROSPERO database (CRD42020216374).

## Introduction

Fibromyalgia (FM) is a chronic condition characterized by generalized body pain, fatigue, sleep disturbance, impaired cognition, and anxiety [1]. The prevalence ranges from 0.2% to 6.6% in the general population [2], causing disability and high direct and indirect costs [3–5]. Patients with FM have higher levels of pain, worse health related quality of life (QoL) and use more medication than patients with other chronic conditions [6, 7]. Although high quality evidence supports the efficacy of some pharmacological interventions, their effects are usually small and not clinically important [8], partially explaining poor patients’ adherence with pharmacotherapy [9–11].

There are many other non-pharmacological interventions for FM [12, 13], and efficacy of some of them has been investigated in systematic reviews [14–20]. Previous pairwise meta-analyses showed effects of exercise [14], acupuncture [15], transcranial magnetic stimulation [20, 21], cognitive behavioral therapy (CBT) [16], transcranial direct current stimulation (tDCS) [22], among others. However, in most cases, this evidence is compromised by the small number of trials available, which leads to imprecision in the effect estimates. Besides, conventional pairwise meta-analysis is limited to the comparison of two interventions at a time and to the existence of previous trials that investigated these interventions directly [23]. Therefore, it is not possible to draw conclusions about the superiority of one form of intervention over the other.

A possible solution to explore all available direct and indirect evidence allowing comparisons of interventions that have never been investigated directly is the network meta-analysis (NMA) [24]. Another advantage of this method is that the incorporation of evidence from indirect comparisons provides more precise estimates for pairs of comparisons where evidence is limited by a small number of low powered trials [25].

To date, only two NMAs have explored multiple intervention comparisons to treat fibromyalgia. Both are outdated NMAs (for example: searches up to 2011 and 2008, respectively) [26, 27]. Both explored pharmacological options and did not investigate interventions that have become more popular in recent years (e.g., TMS and tDCS) [20–22], or others that have shown to be effective in published systematic reviews using conventional pairwise meta-analysis (e.g., acupuncture, resistance exercise, among others) [15, 28]. In addition, previous NMAs did not evaluate the confidence in the effect estimates that became available in recent years (Grading of Recommendations Assessment, Development and Evaluation approach or Confidence in Network Meta-Analysis—CINeMA approach) [29–32], advocated by the PRISMA statement for systematic reviews with NMA [33].

Therefore, the current NMA protocol aims to compare the effectiveness of non-pharmacological interventions commonly used in FM on pain intensity, quality of life and acceptability outcomes. This review will follow the Cochrane recommendations [23], and its results will be reported according to the PRISMA statement for systematic reviews with NMA [33]. Potential sources of heterogeneity, inconsistency and incoherence will be explored, interventions will be ranked according to the probability of being the most effective and accepted, and the confidence in the effect estimates will be assessed using the CINeMA approach [31].

## Materials and methods

A systematic review protocol has been developed and registered with the Open Science Framework (DOI: 10.17605/OSF.IO/7MS25) and registered to PROSPERO database on November 25^th^ 2020 (CRD42020216374). This systematic review protocol was prepared using the Preferred Reporting Items for Systematic Reviews and Meta-Analyses Protocol (PRISMA-P) [34]. We have completed the PRISMA-P checklist (S1 File). We will use the PRISMA-NMA extension statement to structure the contents of the actual systematic review and NMA [33]. Summary of eligibility criteria for Population, Intervention, Comparison, Outcome and Study design (PICOS) is available in Table 1.

**Table 1:** Summary of PICOS eligibility criteria.

	Inclusion criteria
**Population**	Adults with FM diagnosed with any of the ACR criteria (1990, 2010, 2016)
**Intervention**	Non-pharmacological interventions (detailed description in S2 File)
**Comparison**	Non-pharmacological interventions, placebo, sham, no intervention and waiting list
**Outcome**	Pain, quality of life and acceptability
**Study design**	Randomised controlled trials (RCTs)

### Population

#### Inclusion criteria

In this review, we will include participants diagnosed with FM according to the criteria established by the American College of Rheumatology (ACR 1990, 2010 or 2016):

- The ACR 1990 criteria are: (1) history of widespread pain for at least 3 months; and (2) pain in at least 11 out of the 18 tender point sites (i.e. occiput, low cervical, trapezius, supraspinatus, second rib, lateral epicondyle, gluteal, greater trochanter and knee—bilateral in all sites) on digital palpation [35];
- The ACR 2010 criteria are: (1) widespread pain index (WPI, ranges from 0 to 19 points) ≥ 7 points and symptom severity scale (SSS) score (ranges from 0 to 12 points) ≥ 5 points or WPI from 3 to 6 and SSS score ≥ 9 points; (2) symptoms for at least 3 months; and (3) absence of disorders that would otherwise explain the pain [1]; or
- The ACR 2016 criteria are: (1) generalized pain, defined as pain in at least 4 out of the 5 regions; (2) symptoms have been present at a similar level for at least 3 months; (3) WPI ≥ 7 and SSS score ≥ 5 or WPI of 4–6 and SSS score ≥ 9; (4) a diagnosis of FM is valid irrespective of other diagnoses. A diagnosis of FM does not exclude the presence of other clinically important illnesses [36].

#### Exclusion criteria

We will exclude randomised controlled trials (RCTs) not including participants with FM as the primary health condition.

### Interventions

For the purpose of this review, non‐pharmacological interventions refer to management strategies that do not involve the use of nutritional supplements, medications. We pre-defined a set of interventions to be included based on previously published clinical practice guidelines and systematic reviews. Interventions of interest are listed below:

- Acupuncture [13, 15, 37];
- Aerobic exercise training [14];
- Aquatic exercise training [19];
- Balneotherapy and Spa therapy [13, 38];
- Cognitive behavioral therapy, mindfulness meditation therapies, relaxation-based therapies and biofeedback [16, 39];
- Cryotherapy [40];
- Dry needling [37];
- Electrotherapy [41, 42];
- Flexibility exercise [17];
- Heat therapy [40];
- Magnetic field therapy [43];
- Manual therapy [44, 45];
- Massage therapy [46];
- Mixed exercise [47];
- Multicomponent therapy and movement therapies [48];
- Photobiomodulation therapy [49, 50];
- Pilates [51];
- Repetitive transcranial magnetic stimulation [52];
- Resistance exercise [28];
- Therapeutic ultrasound [53];
- Transcranial direct current stimulation [52]; and
- Whole body vibration [18].

The definitions of each intervention node are in the S2 File. We will not exclude RCTs based on frequency, duration or intensity of the delivered intervention. This node-making process is being done *a priori*; however, modifications will be made to adjust for the lack transitivity if necessary. In cases where pre-defined nodes for which they do not have RCTs available or do not connect to the rest of the network will be lumped into other nodes when there is clinical plausibility or will be excluded from the analysis for a given time point. Since transitivity analysis will be conducted before the computation of effect sizes, modifications in node definitions will not be biased by results of NMA.

### Comparator

Another non-pharmacological intervention as specified above or control (i.e., placebo, sham, no intervention or waiting list). In the NMA, two different types of control group, such as placebo/sham and no intervention/waiting list will be combined as one node called “Control”. We understand that placebo/sham groups control for potential nonspecific effect confounders (e.g., patient-therapist interaction and patients’ preferences) plus the natural history of the condition, whereas no intervention and waiting list control only for the natural history of the condition [54]. If inconsistencies are identified in the entire network, in the loops or in the intervention pairs, one of the actions to be taken is to investigate whether the combination of these types of control might have violated the transitivity assumption. In this review, usual care is not going to be considered because the lack of consistency on its definition across trials may potentially violate the transitivity assumption for the NMA. If a trial names a control group as “usual care” or “standard care” but it clearly states that participants did not receive any active intervention that begun during the study, we will include it considering as "no intervention".

### Outcomes

We will include studies that measured at least one of the following outcomes:

#### Primary outcomes

*Pain intensity*. we will preferentially extract data measured with numerical rating scales (NRS). When NRS is not available, we will use data measured with other instruments in the following order: visual analog scale (VAS), verbal rating scale (VRS) or Likert scales. Instruments that evaluate multidimensional aspects of pain (e.g., Brief Pain Inventory—BPI), (short-form McGill Pain Questionnaire—SF/MPQ) will be considered only if it presents separate results for pain intensity measured with its NRS or VAS. In this review, assessments of multidimensional aspects or interference of pain will not be considered. Published trials commonly report multiple assessments of pain intensity (e.g., worst pain, average pain, pain in the last week). We will preferably extract data of average pain in the last 24 hours and pain at rest. The instrument used to assess pain intensity will be reported in the characteristics of the included trials.

*Quality of life (QoL)*. For QoL, we will only consider trials that have used the Fibromyalgia Impact Questionnaire (FIQ). This is a valid instrument and was chosen because it is able to assess the characteristic symptoms of this health condition such as pain, depression, fatigue, stiffness and anxiety [55].

#### Secondary outcomes

*Acceptability*. Treatment discontinuation due to any reason will be used to assess acceptability. It will be measured by the number of participants who withdrew due to any reason out of the total number of participants randomly assigned to each treatment arm. This data will be collected at the same time points considered for the assessment of pain intensity and/or QoL.

### Study designs

Parallel group RCTs, cross over RCTs and cluster RCTs that include at least two arms with different forms of interventions of interest listed above (topic "*Interventions*"). Quasi-RCTs will not be included because allocation procedures do not satisfy this criterion (i.e., allocation by hospital record number, birth date or alternation).

### Search strategy

Search strategies will be conducted in: MEDLINE, Cochrane Library, EMBASE, AMED, PsycINFO and PEDro without language or date restrictions (S3 File). In addition, we will hand search for potentially relevant full texts included in published systematic reviews on the topic and reference lists of the included trials.

### Study selection

After electronic searches, the retrieved references will be exported to a reference manager (i.e., Endnote®) file and duplicates will be removed. Two independent reviewers (MS and RM) will screen titles and abstracts, and assess potential full texts. Full texts fulfilling the eligibility criteria will be included in the review. If necessary, authors will be contacted by email to clarify information. Three attempts by email in-between seven days will be made. If authors do not answer, the trial will be excluded and the reasons for exclusion will be reported in a PRISMA flowchart diagram. Between-reviewer discrepancies will be resolved by a third reviewer (VCO).

### Data extraction

The following data will be extracted:

Methods: study design (i.e., parallel group, cross-over or cluster RCT), number of study centers and location, study setting, and year of publication;

Participants: sample size, mean age, sex, disease duration, participants’ pain score at baseline, comorbidities, use of medications, diagnostic criteria, inclusion and exclusion criteria, number of randomised, analysed, withdrawal in each group, and whether data was analysed in accordance with the intention-to-treat principle;

Interventions: mode, duration, frequency, intensity and main characteristics;

Outcomes: primary (i.e., pain intensity and QoL) and secondary (i.e., acceptability) outcomes of interest, instruments, range score, time points. Where possible we will extract data at the arm level, not summary effects (post-intervention data and not the effect over time); and

Notes: language of publication, contact with authors for additional information.

For continuous outcomes (i.e., pain intensity and QoL), means, standard deviations (SDs) and sample sizes of groups will be extracted for immediate-, short- and long-term effects. We will consider immediate-term effects follow-ups from randomisation up to 2 weeks, short-term effects follow-ups over 2 weeks after randomisation up to 12 weeks and long-term effects follow-ups over 12 weeks after randomisation. If more than one time point is available within the same follow-up, we will consider the closest to the end of the intervention. Mean changes from baseline and SDs will be extracted if post-intervention scores are not available. For the calculation of Relative Risks (RRs) as indicator of treatment acceptability, the number of dropouts between beginning and end of the treatment will be extracted. If dropout rates are not reported, the difference between the number of patients at the beginning and at the end of treatment will be used. Acceptability data will be estimated by summarizing the evidence from the collection of trials that assessed pain intensity and QoL outcome separately. The definition and decision rules regarding data collection by time point will be the same as those presented above for pain intensity and QoL.

When trials include two or more arms comprehending different doses, frequency or intensity of the same intervention, we will combine outcome data following the Cochrane recommendations. In trials where SDs are not available, they will be imputed from the standard error, confidence interval, p-value, range values, interquartile interval or from other similar trials included, following the recommendations [23]. When data is only presented in graphs it will be extracted using the Ycasd software [56]. When imputations are not possible as described above, the corresponding authors will be contacted by email as described previously. If the authors do not answer, the study will be excluded from quantitative analysis but will be included in the review. Our primary interest is the effect of assignment to intervention, so we will seek results for the intention‐to‐treat (as randomised) population. If data are missing due to participant dropout, we will use reported results for participants that completed the study. Data extraction will be conducted by two independent authors (MS and RM) using previously prepared electronic forms. Discrepancies will be resolved by a third author (VCO).

For cross-over RCTs, we will only consider results from the first randomisation period to avoid carry-over effects. We do not expect to find any cluster-RCT, but if available, we will include them and data will be extracted following the recommendations in Cochrane Handbook [23].

### Risk of bias assessment using the Cochrane risk of bias tool (RoB 2)

Risk of bias of the included trials will be assessed using the Cochrane risk of bias tool (RoB 2) [57]. The following five domains will be assessed: (1) bias arising from the randomisation process; (2) bias due to deviations from intended interventions; (3) bias due to missing outcome data; (4) bias in measurement of the outcome; (5) bias in selection of the reported result. We will use the algorithms described in the instrument for classification of each domain as: (1) low risk of bias; (2) some concerns; and (3) high risk of bias. The judgement of the overall risk of bias of the included trial will follow the rule: (1) low risk of bias, low risk of bias for all domains; (2) some concerns, some concerns for at least one domain but no high risk of bias in any domain; (3) high risk of bias, high risk of bias in at least one domain or have some concerns for multiple domains in a way that substantially lowers confidence in the result.

The evaluation of the domain ‘bias due to deviations from the intended interventions’ will be done with an interest in quantifying the effect of the attribution to the interventions in the baseline, regardless of whether the interventions are received as intended.

In multi-arm trials that contains more than two groups of interest, the risk of bias will be assessed for each possible comparison between pairs of interventions. For instance, in a trial that investigated interventions A, B and C, the risk of bias will be assessed for the comparison of A and B, A and C, and B and C. Moreover, assessments will be made for each outcome and time point separately. Between-reviewer discrepancies will be resolved by a third reviewer (VCO).

The results of this assessment will be used for downgrading the quality of the evidence using the CINeMA framework and for the sensitivity analysis (as described below).

### Strategy for data synthesis

For each pairwise comparison between the different interventions, we will present mean differences (MDs) for pain intensity and QoL outcomes and RRs for acceptability outcome, both with its respective 95% confidence intervals (CIs). We will assess the presence of clinical heterogeneity within each pairwise comparison by comparing the trial and study population characteristics across all eligible trials. Standard pairwise meta-analyses will be performed using a random effects model in STATA version 16 (StataCorp LLC, College Station, TX) for all comparisons with at least two trials. Statistical heterogeneity within each pairwise comparison will be assessed using the I^2^ statistics, where an I^2^ ≥ 50% indicates heterogeneity [23].

Assumption of transitivity will be evaluated by comparing the distribution of study and population characteristics that could act as effect modifiers across the different pairwise comparisons. Random effects frequentist NMA will be conducted if there is no evidence for important intransitivity. We will perform analyses using the *mvmeta* command and network suite in STATA. If there are issues with transitivity, we will go back to data extraction and re-check the inclusion criteria of the trials, the description of the intervention groups and comparators, and also the instruments used to measure pain and quality of life outcomes. If necessary, we will break an intervention node into two nodes or we will lump two nodes (if there is clinical plausibility for this decision) into one. Excluding studies or an intervention node will always be our last option.

In NMA, we will assume a common estimate for the heterogeneity variance across the different comparisons. The assumption of coherence will be evaluated statistically using both local and global approach. Specifically, side-splitting method will be used to evaluate incoherence for every comparison with available direct evidence and the design-by-treatment interaction model to tests incoherence in the entire network. In case of significant incoherence, its possible sources will be investigated by the assessment of an uneven distribution of effect modifiers across groups of trials that compare different interventions. Therefore, the distribution of clinical and methodological variables that we suspect may be potential sources of either heterogeneity or inconsistency in each comparison-specific group of trials will be investigated.

The competing interventions will be ranked using the P-score, which is the frequentist analogue of surface under the cumulative ranking curve (SUCRA) for the outcomes of interest (i.e., pain intensity, QoL and acceptability) at immediate-, short- and long-terms [58].

#### Assessment of publication bias

Publication bias will be assessed by visual inspection of funnel plots and funnel plot asymmetry tests for pair‐wise meta‐analysis, cld validity conditions are met (i.e., low heterogeneity, 10 or more studies with at least one with significant results, and a ratio of the maximal to minimal variance across studies greater than four, and comparison‐adjusted funnel plots for NMA). We will assess small‐study effects and related reporting bias for primary outcomes. In cases of evidence of small‐trial effects, sensitivity analyses according to a regression‐based adjustment model for every comparison with direct evidence available will be performed.

#### Investigation heterogeneity and inconsistency

If we find significant heterogeneity or inconsistency (or both) between comparisons, we will explore its possible sources. We will investigate the distribution of clinical and methodological characteristics that can act as effect modifiers between comparisons and, if sufficient trials (i.e., at least 10 trials analysed) are available, we will perform network meta-regression. For FM, potential effect modifiers that will be explored are:

- Diagnostic criteria (i.e., ACR 1990; ACR 2010; ACR 2016);
- When the presence of any comorbidity is part of the study’s inclusion criteria;
- Characteristic of the intervention (i.e., mode, dosage, intensity);
- Use of medications for management of FM symptoms; and
- Duration of the intervention within each timepoint (i.e., immediate-, short- and long-term).
- Participants’ pain score at baseline.
- Control group types (e.g., placebo/sham and no intervention/waiting list).

#### Assessment of quality of evidence using Confidence in Network Meta‐analysis (CINeMA)

The confidence in the results from NMA will be assessed using the CINeMA framework [31]. This framework is broadly based on the GRADE framework and considers six domains: (i) within-study bias; (ii) reporting bias; (iii) indirectness; (iv) imprecision; (v) heterogeneity; and (vi) incoherence. Each of these domains can be judged at three levels (no concerns, some concerns, or major concerns). The range of equivalence used for the judgements of imprecision, heterogeneity and incoherence will be defined according to the minimal clinically important change (MCID) available for people with FM (i.e., 2 points on a 11-points NRS for pain intensity and 14 point in a 101-points FIQ scale for QoL) [59, 60]. For the within-study bias domain, the weighted average of the risk of bias as calculated by CINeMA will be used. Two authors (MS and RM) will independently make the judgements about quality of the evidence (high, moderate, low, or very low), and discrepancies will be resolved by a third reviewer (VCO).

## Discussion

Our NMA intends to evaluate the comparative efficacy and acceptability of non-pharmacological interventions endorsed in guidelines for the treatment of FM. To date, we are aware of only two NMAs that have investigated conservative treatments in the management of FM published [26, 27], and of a NMA protocol published in 2013 that proposed to evaluate all treatments available for this health condition, but the results of the study have not been published [61].

One of the strengths of our NMA will be that the node-making process for the interventions to be analysed is being defined *a priori* and based on definitions previously published in Cochrane reviews (with the exception of multicomponent therapy) [48], in order to make this step more transparent and based on clinical arguments. This is important since it has been reported in the literature that less than 10% of NMAs report how this process happens during the conduct of the review [62]. In addition, we will analyse the risk of bias of the included trials with RoB 2 [57], and this assessment will be made for each comparison between studies and for each outcome and timepoint in each study and we will rate the confidence of the evidence that contributes to the estimation of interventions included in the network through the CINeMA approach [31]. Another strong point of our NMA is that we intend to assess and rank the acceptability of interventions in the management of FM, since it has been reported in the literature that this is a problem in this health condition [63].

FM is a health condition that is difficult to manage, patients often feel that their complaints are ignored by health professionals and also face difficult experiences in health systems [64]. Although much has been tried in relation to non-pharmacological treatment options for the management of FM, the available interventions have effect sizes that are only modest and the recommendations for their use are weak in most cases [13], in addition, the available effect size estimates are inaccurate and few RCTs make direct comparisons between two forms of intervention that prove to be effective. This NMA will be of great importance for patients, health professionals, researchers, insurers and for public health policies, because it will provide a classification of non-pharmacological interventions according to their efficacy and acceptability, in order to assist in clinical decision making.

## Supporting information

S1 File Research checklist—PRISMA-P (Preferred Reporting Items for Systematic review and Meta-Analysis Protocols) 2015 checklist: Recommended items to address in a systematic review protocol. (DOCX)

S2 File Definitions of each intervention node. (DOCX)

S3 File Search strategy. (DOCX)
