# Supplementary material for: Comparative efficacy and acceptability of non-pharmacological interventions in fibromyalgia: Protocol for a network meta-analysis
Source: PLoS One. 2022 Oct 3;17(10):e0274406. doi: 10.1371/journal.pone.0274406 (PMC9529083; doi:10.1371/journal.pone.0274406)
Supplement: S2 File — (DOCX) [file pone.0274406.s002.docx]

**S2 File**. Definitions of each intervention node.

Intervention classes defined according to interventions commonly used in the management of fibromyalgia. All intervention nodes will be investigated, regardless of frequency, duration or intensity. Definitions are based on previously published randomised controlled trials and systematic reviews that investigated the efficacy of interventions in fibromyalgia:

**1. Acupuncture**

Acupuncture will be defined as “use of traditional acupuncture theory, with needles being inserted into classic meridian points, extra points or ah-shi points.” It will be excluded studies in which the acupuncture treatment did not involve needling (acupressure or laser acupuncture) or which have been delivered in association with another component (electroacupuncture) [1–3].

**2. Aerobic exercise training**

Aerobic exercise will be defined according to the guidelines of the American College of Sports Medicine, which can also be called cardiorespiratory exercise. It can include a wide range of activities, such as walking, running, cycling and dancing, being performed at submaximal intensity and lasting from minutes to hours [4].

**3. Aquatic exercise training**

Aquatic exercise will be defined according to the Chartered Society of Physiotherapists as a therapeutic program using the properties of water to improve function. We will consider only those aquatic exercise interventions that involve exercise in the water for 50% or more of the time [5].

**4. Balneotherapy or Spa therapy**

Balneotherapy will be defined through the use of hot water as a form of treatment in order to relieve pain, relax muscles and decrease stiffness, and can be delivered in various ways, such as mineral baths, sulfur baths, mud packs. SPA therapy will be included as an equivalent intervention and can be performed using jet streams [2,6].

**5. Cognitive behavioral therapy, mindfulness meditation therapies, relaxation-based therapies and biofeedback**

**5.1. Cognitive behavioral therapy (CBT)** – It will be defined as an intervention that aims to modify negative thoughts about pain and promote behavioral changes in order to improve function and develop coping strategies to deal with pain. We will consider the following techniques: traditional CBT, operant therapy, self-management educational programs and acceptance-based CBTs programs [7]. Besides, mindfulness meditation therapies, relaxation-based therapies and biofeedback will be also considered CBT:

**5.2. Mindfulness meditation therapies -** It will be defined as a way of looking at the world and being aware of the present moment without making judgments [8].

**5.3. Relaxation-based therapies** - It will be defined as techniques that aim to calm the mind and relax the body, such as breathing techniques, visual imagery, guided imagery, progressive muscle relaxation [8].

**5.4. Biofeedback -** It will be defined as a technique that connects the individual to electrical devices that provide information through visual and auditory feedback in order to help the individual to relax and gain more control over their bodies [8].

**6. Cryotherapy**

Cryotherapy will be defined by applying cold, ice packs or massage with ice over painful areas or acupoints, with the objective of relieving pain [9].

**7. Dry needling**

Dry needling will be defined by applying needles to myofascial trigger points identified by palpation [1].

**8. Electrotherapy**

Electrotherapy will be defined as a treatment that implements non-invasive electrical current to promote pain relief and improve function. This may include: transcutaneous electrical nerve stimulation (TENS), interferential current, galvanic current. Iontophoresis will not be considered because the medicinal ion component is supposed to have an additional effect to the electrical current [10,11].

**9. Flexibility exercise**

Flexibility exercise will be defined as an exercise that aims to gain or maintain the range of motion of joint and muscle structures [12].

**10. Heat therapy**

Heat therapy will be defined through the application of hot packs, superficial heat, in order to decrease pain and/or stiffness, increase mobility, help to relax the muscles [9].

**11. Magnetic field therapy**

It will be defined as an intervention that consists of the delivery of pulsed electromagnetic fields or pulsed electrical stimulation through electrodes placed directly on the body in order to temporarily relieve pain [13].

**12. Manual therapy**

We will consider manipulation and/or mobilization techniques as manual therapy. Manipulation will be defined through techniques that involve the application of a force at high speed and low amplitude directed at specific spine segments. Mobilizations will be defined as techniques that involve passive movement of low-degree/velocity, in a small or large amplitude. Neuromuscular mobilization techniques will also be considered within the control and range of motion of the patient [14,15].

**13. Massage therapy**

Massage will be defined as any soft tissue manipulation done with the hands or with auxiliary devices. The massage may have been applied to any part of the body [16].

**14. Mixed exercise**

Mixed training will be defined as an intervention that contain at least two of the following modalities of exercise (i.e. aerobic, resistance and flexibility). Each type of exercise had to contribute as a significant part of the exercise intervention. Other types of exercise (e.g., co‐ordination, balance, and relaxation involving voluntary muscle contractions), could also contribute to the intervention [17].

**15. Multicomponent therapy and movement therapies**

**15.1.** Multicomponent therapy will be defined as an intervention that involves the combination of any modality exercise associated with at least one psychological therapy [18].

**15.2.** Movement therapies: yoga, tai chi, qi-gong [8].

**16.** **Photobiomodulation therapy**

Red or near infrared light (low-power laser [LLLT] or light emitting diode [LED]) used modulate inflammation, accelerate healing, or reduce pain and discomfort will be considered as photobiomodulation [19]. Trials that have investigated the effects of all types of LLLT (Classes I, II and III) will be considered [20].

**17. Pilates**

Pilates will be considered as an intervention that has used the principles of this method during the exercise, such as: posture, breathing, flow, precision, control, concentration and centralization (or at least three of these components) [21].

**18. Repetitive transcranial magnetic stimulation (rTMS)**

rTMS will be defined as an intervention that involves stimulation of the cerebral cortex through a coil applied directly to the scalp. Electric currents are applied to neurons through the use of rapidly changing magnetic fields. These currents are applied to the cerebral cortex in order to make changes in brain activity [22].

**19. Resistance exercise**

Resistance exercise will be defined as an intervention that aims to gain resistance, strength, muscle power or a combination of these. We will not establish a minimum duration of specific intervention. Resistance during training can be applied through free weights, elastic bands, weight machines, calisthenics (the use weight of a body segment or segments moving against gravity as the load for the exercise) [23].

**20. Therapeutic ultrasound**

Will be included any form of therapeutic ultrasound delivery (e.g., continuous or pulsed, at any frequency, intensity, and duration). In this review, phonophoresis (i.e., the use of ultrasound to enhance the delivery of topically applied drugs) or extracorporeal ultrasonic treatment (i.e., derivative of lithotripsy) will not be considered [24].

**21. Transcranial direct current stimulation (tDCS)**

tDCS will be defined as an intervention that consists of the application of an electric current in the cerebral cortex with the aim of modulating brain activity [22].

**22. Whole body vibration (WBV)**

WBV will be defined as an intervention in which the individual exercises on an oscillatory platform (moving or maintaining a standing position). We will consider interventions regardless of the frequency, duration or intensity of the exercise session [25].

**REFERENCES**

1. Furlan AD, Van Tulder M, Cherkin D, Tsukayama H, Lao L, Koes B, et al. Acupuncture and dry-needling for low back pain: An updated systematic review within the framework of the cochrane collaboration. Spine. 2005. pp. 944–963. doi:10.1097/01.brs.0000158941.21571.01

2. Macfarlane GJ, Kronisch C, Dean LE, Atzeni F, Häuser W, Fluß E, et al. EULAR revised recommendations for the management of fibromyalgia. Ann Rheum Dis. 2017;76: 318–328. doi:10.1136/annrheumdis-2016-209724

3. Deare JC, Zheng Z, Xue CCL, Liu JP, Shang J, Scott SW, et al. Acupuncture for treating fibromyalgia. Cochrane Database of Systematic Reviews. John Wiley and Sons Ltd; 2013. p. CD007070. doi:10.1002/14651858.CD007070.pub2

4. Bidonde J, Busch AJ, Schachter CL, Overend TJ, Kim SY, Góes SM, et al. Aerobic exercise training for adults with fibromyalgia. Cochrane database Syst Rev. 2017;6: CD012700. doi:10.1002/14651858.CD012700

5. Bidonde J, Busch AJ, Webber SC, Schachter CL, Danyliw A, Overend TJ, et al. Aquatic exercise training for fibromyalgia. Cochrane Database Syst Rev. 2014;2014. doi:10.1002/14651858.CD011336

6. Verhagen AP, Bierma-Zeinstra SM, Boers M, Cardoso JR, Lambeck J, de Bie R, et al. Balneotherapy (or spa therapy) for rheumatoid arthritis. Cochrane Database Syst Rev. 2015;2015. doi:10.1002/14651858.CD000518.pub2

7. Bernardy K, Klose P, Busch AJ, Choy EHS, Häuser W. Cognitive behavioural therapies for fibromyalgia. Cochrane Database Syst Rev. 2013;2017. doi:10.1002/14651858.CD009796.pub2

8. Theadom A, Cropley M, Smith HE, Feigin VL, Mcpherson K. Mind and body therapy for fibromyalgia. Cochrane Database of Systematic Reviews. John Wiley and Sons Ltd; 2015. doi:10.1002/14651858.CD001980.pub3

9. Brosseau L, Yonge K, Welch V, Marchand S, Judd M, Wells GA, et al. Thermotherapy for treatment of osteoarthritis. Cochrane Database Syst Rev. 2003;2003. doi:10.1002/14651858.cd004522

10. Johnson MI, Claydon LS, Herbison GP, Jones G, Paley CA. Transcutaneous electrical nerve stimulation (TENS) for fibromyalgia in adults. Cochrane Database Syst Rev. 2017;2017. doi:10.1002/14651858.CD012172.pub2

11. Page MJ, Green S, Kramer S, Johnston R V., Mcbain B, Buchbinder R. Electrotherapy modalities for adhesive capsulitis (frozen shoulder). Cochrane Database of Systematic Reviews. 2014. doi:10.1002/14651858.CD011324

12. Kim SY, Busch AJ, Overend TJ, Schachter CL, van der Spuy I, Boden C, et al. Flexibility exercise training for adults with fibromyalgia. Cochrane Database Syst Rev. 2019;2019. doi:10.1002/14651858.CD013419

13. Li S, Yu B, Zhou D, He C, Zhuo Q, Hulme JM. Electromagnetic fields for treating osteoarthritis. Cochrane Database of Systematic Reviews. John Wiley and Sons Ltd; 2013. doi:10.1002/14651858.CD003523.pub2

14. Gross A, Langevin P, Burnie SJ, Bédard-Brochu MS, Empey B, Dugas E, et al. Manipulation and mobilisation for neck pain contrasted against an inactive control or another active treatment. Cochrane Database of Systematic Reviews. John Wiley and Sons Ltd; 2015. doi:10.1002/14651858.CD004249.pub4

15. Schulze NB, Salemi M de M, de Alencar GG, Moreira MC, de Siqueira GR. Efficacy of Manual Therapy on Pain, Impact of Disease, and Quality of Life in the Treatment of Fibromyalgia: A Systematic Review. Pain Physician. 2020;23: 461–476. Available: https://pubmed.ncbi.nlm.nih.gov/32967389/

16. Furlan AD, Giraldo M, Baskwill A, Irvin E, Imamura M. Massage for low-back pain. Cochrane Database of Systematic Reviews. John Wiley and Sons Ltd; 2015. doi:10.1002/14651858.CD001929.pub3

17. Bidonde J, Busch AJ, Schachter CL, Webber SC, Musselman KE, Overend TJ, et al. Mixed exercise training for adults with fibromyalgia. Cochrane Database of Systematic Reviews. John Wiley and Sons Ltd; 2019. doi:10.1002/14651858.CD013340

18. Arnold B, Häuser W, Arnold M, Bernateck M, Bernardy K, Brückle W, et al. Multimodale Therapie des Fibromyalgiesyndroms. Der Schmerz. 2012;26: 287–290. doi:10.1007/s00482-012-1173-1

19. Hamblin MR. Mechanisms and applications of the anti-inflammatory effects of photobiomodulation. AIMS Biophysics. American Institute of Mathematical Sciences; 2017. pp. 337–361. doi:10.3934/biophy.2017.3.337

20. Yeh SW, Hong CH, Shih MC, Tam KW, Huang YH, Kuan YC. Low-level laser therapy for fibromyalgia: A systematic review and meta-analysis. Pain Physician. 2019;22: 241–254. Available: https://pubmed.ncbi.nlm.nih.gov/31151332/

21. Yamato TP, Maher CG, Saragiotto BT, Hancock MJ, Ostelo RWJG, Cabral CMN, et al. Pilates for low back pain. Cochrane Database of Systematic Reviews. John Wiley and Sons Ltd; 2015. doi:10.1002/14651858.CD010265.pub2

22. O’Connell NE, Wand BM, Marston L, Spencer S, DeSouza LH. Non-invasive brain stimulation techniques for chronic pain. Cochrane Database Syst Rev. 2014;2014. doi:10.1002/14651858.CD008208.pub3

23. Busch AJ, Webber SC, Richards RS, Bidonde J, Schachter CL, Schafer LA, et al. Resistance exercise training for fibromyalgia. Cochrane Database Syst Rev. 2013;2013. doi:10.1002/14651858.CD010884

24. Rutjes AW, Nüesch E, Sterchi R, Jüni P. Therapeutic ultrasound for osteoarthritis of the knee or hip. Cochrane database Syst Rev. 2010; CD003132. doi:10.1002/14651858.CD003132.pub2

25. Bidonde J, Busch AJ, van der Spuy I, Tupper S, Kim SY, Boden C. Whole body vibration exercise training for fibromyalgia. Cochrane Database Syst Rev. 2017;2017. doi:10.1002/14651858.CD011755.PUB2
