# Supplementary material for: Comparative efficacy and acceptability of non-pharmacological interventions in fibromyalgia: Protocol for a network meta-analysis
Source: PLoS One. 2022 Oct 3;17(10):e0274406. doi: 10.1371/journal.pone.0274406 (PMC9529083; doi:10.1371/journal.pone.0274406)
Supplement: S3 File — (DOCX) [file pone.0274406.s003.docx]

**S3 File.** Search strategy.

**OVID (MEDLINE, COCHRANE, EMBASE, AMED, PSYCINFO)**

1. randomised controlled trial*.mp. [mp=ab, hw, ti, tx, kw, ct, ot, sh, tc, id, tm, tn, dm, mf, dv, fx, nm, kf, px, rx, ui, sy]

2. Randomized Controlled Trial.mp. [mp=ab, hw, ti, tx, kw, ct, ot, sh, tc, id, tm, tn, dm, mf, dv, fx, nm, kf, px, rx, ui, sy]

3. random allocation.mp. [mp=ab, hw, ti, tx, kw, ct, ot, sh, tc, id, tm, tn, dm, mf, dv, fx, nm, kf, px, rx, ui, sy]

4. Comparative Stud*.mp. [mp=ab, hw, ti, tx, kw, ct, ot, sh, tc, id, tm, tn, dm, mf, dv, fx, nm, kf, px, rx, ui, sy]

5. Controlled Clinical Trial*.mp. [mp=ab, hw, ti, tx, kw, ct, ot, sh, tc, id, tm, tn, dm, mf, dv, fx, nm, kf, px, rx, ui, sy]

6. double-blind method*.mp. [mp=ab, hw, ti, tx, kw, ct, ot, sh, tc, id, tm, tn, dm, mf, dv, fx, nm, kf, px, rx, ui, sy]

7. single-blind method*.mp. [mp=ab, hw, ti, tx, kw, ct, ot, sh, tc, id, tm, tn, dm, mf, dv, fx, nm, kf, px, rx, ui, sy]

8. Clinical Trial*.mp. [mp=ab, hw, ti, tx, kw, ct, ot, sh, tc, id, tm, tn, dm, mf, dv, fx, nm, kf, px, rx, ui, sy]

9. crossover stud*.mp. [mp=ab, hw, ti, tx, kw, ct, ot, sh, tc, id, tm, tn, dm, mf, dv, fx, nm, kf, px, rx, ui, sy]

10. 1 or 2 or 3 or 4 or 5 or 6 or 7 or 8 or 9

11. fibromyalgia.mp. [mp=ab, hw, kw, ti, ot, tx, ct, sh, tc, id, tm, tn, dm, mf, dv, fx, nm, kf, px, rx, an, ui, sy]

12. 10 and 11

**PEDro**

Abstract & Title: Fibromyalgia

Therapy: not applicable

Problem: not applicable

Body Part: not applicable

Subdiscipline: not applicable

Topic: not applicable

Method: not applicable

Author/Association: not applicable

Title Only: not applicable

Source: not applicable

Published Since: not applicable

New records added since: not applicable

Score of at least: not applicable
